# Association of Macronutrients Composition, Physical Activity and Serum Androgen Concentration in Young Women with Polycystic Ovary Syndrome

**DOI:** 10.3390/nu14010073

**Published:** 2021-12-24

**Authors:** Małgorzata Mizgier, Rafał Watrowski, Justyna Opydo-Szymaczek, Elżbieta Jodłowska-Siewert, Giovanni Lombardi, Witold Kędzia, Grażyna Jarząbek-Bielecka

**Affiliations:** 1 Dietetic Department, Faculty of Physical Culture, Poznan University of Physical Education, 66-400 Gorzów Wielkopolski, Poland; 2 Faculty of Medicine, University of Freiburg, 79106 Freiburg, Germany; Rafal.Watrowski@gmx.at; 3 Department of Pediatric Dentistry, Poznan University of Medical Sciences, 60-812 Poznan, Poland; jopydo@ump.edu.pl; 4 Department of Computer Science and Statistics, Poznan University of Medical Sciences, 60-806 Poznan, Poland; youleadmeastray@gmail.com; 5 Laboratory of Experimental Biochemistry and Molecular Biology, IRCCS Istituto Ortopedico Galeazzi, 20161 Milano, Italy; giovanni.lombardi@grupposandonato.it; 6 Department of Athletics, Strength and Conditioning, Poznań University of Physical Education, 61-871 Poznań, Poland; 7 Department of Perinatology and Gynecology, Division of Developmental Gynecology and Sexology, Poznan University of Medical Sciences, 60-535 Poznan, Poland; witold.kedzia@poczta.fm (W.K.); grajarz@o2.pl (G.J.-B.)

**Keywords:** adolescents, adolescent girls, hyperandrogenism, polycystic ovary syndrome, macronutrients, diet, nutrition, physical activity

## Abstract

The roles of dietary macronutrients and physical activity (PA) in patients with PCOS have not been sufficiently reported, especially in adolescent girls. To address this knowledge gap, we evaluated the associations between serum concentrations of total testosterone (tT), free testosterone (fT), androstenedione (A), dehydroepiandrosterone-sulfate (DHEA-S), sex hormone-binding globulin (SHBG) and dietary macronutrients intake as well as different types and levels of PA. The study population consisted of 96 girls of Caucasian ancestry, aged 14–18 years: 61 participants with polycystic ovary syndrome (PCOS) and 35 healthy controls. Serum tT, fT, A, DHEA-S, and SHBG were determined in fasting blood. Macronutrient intake and PA levels were assessed by using the three-day food record method and the Beliefs and Eating Habits Questionnaire (KomPAN), respectively. We found several positive correlations between dietary macronutrients such as total fat, saturated fatty acids (SFA), monounsaturated (MUFA) and polyunsaturated fatty acids (PUFA), and hormonal parameters across the entire cohort and in healthy girls. A positive correlation between SHBG and total protein consumption as well as an inverse correlation between SHBG and carbohydrate intake could be determined. No correlation between androgens and macronutrients was found in the PCOS group. In contrast, we observed an inverse correlation between androgen concentrations (except of DHEA-S) and “work/school” and/or “leisure time” PA only in PCOS patients. Moreover, the hormone levels differed according to PA intensity. In conclusion, the impact of diet and PA was strikingly different in adolescents with and without PCOS. These findings indicate that disturbed hormonal homeostasis in PCOS, at least in the youngest patients, likely “overtrump” dietary influences, and otherwise, PA offers a therapeutic potential that requires further evaluation of the long-term effects in randomized studies. (ClinicalTrial.gov Identifier: NCT04738409.)

## 1. Introduction

Polycystic ovary syndrome (PCOS) is the most common endocrinopathy, affecting 5–13% of women of reproductive age [1,2,3]. In adolescents, depending on the criteria used, PCOS is diagnosed in 3.4%, 8% and 11% (based on criteria of the National Institutes of Health, Androgen Excess and Polycystic Ovary Syndrome Society, and Rotterdam, respectively) [1]. It is also the most frequently diagnosed endocrine disorder in obese women [4,5].

The classical diagnostic criteria of PCOS are based on (and limited to) hyperandrogenemia/hyperandrogenism, chronic anovulation and/or polycystic ovarian morphology (PCOM) on ultrasound. However, PCOS comprises a much wider spectrum of endocrine and metabolic alterations, including insulin resistance (IR), disturbed gonadotropic and neuropeptide secretion, obesity, type 2 diabetes (T2DM), atherogenic dyslipidemia, and increased overall cerebrovascular morbidity [6]. For instance, insulin resistance is estimated at 30% of lean and 70% of obese women with PCOS, whereas the prevalence of impaired glucose tolerance (IGT) and T2DM in PCOS patients is reported at 23–35% and 4–10%, respectively. Furthermore, lipid abnormalities are diagnosed in 70% of women with PCOS [7,8,9,10,11]. IR contributes to the stimulation of the ovaries and/or adrenal glands to increase the production of androgens [12,13]. It is noteworthy that the sonographic PCOM that gave the syndrome its name is not a mandatory criterion in adolescents due to frequent multi-follicular appearance of ovaries in this life stage and less reliable transabdominal ultrasound in virgin girls [14]. While infertility associated with PCOS is often the most troublesome symptom in adult patients, the burden of disease in adolescents is mainly determined by clinical hyperandrogenism (seborrhea, acne, hirsutism) and overweight [14,15,16].

As mentioned above, more than half of women with PCOS live with overweight and obesity [17]. Abdominal obesity, in turn, is linked to increased IR, alterations of carbohydrate and lipid metabolism, chronic inflammation, and increased risk of developing hypertension [5]. In general, the metabolic profile observed in PCOS is very similar to that of metabolic syndrome [5]. Notably, normal-weight patients diagnosed with PCOS present similar metabolic risks as obese women [5,18]. The goals of PCOS treatment comprise the normalization of androgen excess, restoration of normal ovulation and fertility, and also the prevention of delayed cardiovascular (e.g., coronary disease), metabolic (e.g., obesity, T2DM, metabolic syndrome) or oncological (e.g., endometrial cancer) comorbidities. Therefore, lifestyle changes including increased physical activity (PA) and diet modification play an important role in the management of PCOS [19,20,21,22].

Diagnosing and treating PCOS in adolescents is challenging. The classic diagnostic criteria are confused in adolescence by frequent menstrual disorders due to the immaturity of the hypothalamic–pituitary–ovarian axis, the lack of well-validated criteria for hyperandrogenism (except for hirsutism) and the limited informative value of sonographic criteria (PCOM). In addition, long-term pharmacological interventions during puberty are ultimately less accepted by both patients and doctors [14,15]. Hence, evidence-based behavioral and nutritional interventions may be of interest. However, the non-pharmacological interventions were predominantly studied with a focus on weight reduction. Healthy diet and weight normalization are beneficial regardless of the PCOS diagnosis. Since hyperandrogenism is responsible for the most common and troublesome clinical signs of PCOS in adolescents (acne, hirsutism, etc.), the influence of lifestyle factors and macronutrient dietary intake on androgen excess deserves attention. We believe that diet and physical activity could improve PCOS properties not only through weight loss; however, the biological plausibility of these assumptions has not been adequately proven [23,24,25,26,27,28,29].

To address the aforementioned knowledge gaps, we aimed to assess the relationship between the consumption of dietary macronutrients, PA, and the concentration of androgens. Furthermore, we aimed to determinate the relationship between the level of PA and androgen status.

## 2. Materials and Methods

### 2.1. Study Design and Population

This case–control study was carried out from 2018 to 2020 at the Department of Gynecology and Perinatology, Gynecology and Obstetrics Hospital of the University of Medical Sciences in Poznań, Poland. Sixty-one Caucasian girls aged 14–18 years fulfilling the ESHRE/ASRM Rotterdam 2003 PCOS criteria [30], and thirty-five young, same-aged women without PCOS were included in the study. The “Rotterdam” 2003 PCOS criteria require the presence of two out of the following three findings for the diagnosis of PCOS: signs of clinical or biochemical hyperandrogenism (hirsutism with moderate to severe acne, and/or the elevation of total testosterone or free testosterone levels), and/or chronic ovulatory dysfunction (based on oligomenorrhea defined as bleeding episodes occurring less than 8 times per year or secondary amenorrhea), and/or PCOM on ultrasound (at least 12 follicles in each ovary each measuring 2–9 mm in diameter and/or ovarian volume >10 mL), after exclusion of secondary causes [30]. The exclusion criteria were defined as follows: any systemic disease, thyroid dysfunction, diabetes, congenital adrenal hyperplasia, Cushing syndrome, hyperprolactinemia suggestive of pituitary adenoma and androgen-secreting tumors, continuous medications, use of hormonal therapies or antibiotics in the past three months, vitamin or supplement use, regular alcohol consumption, and smoking. The participants were enrolled in the study through the use of the non-probability sampling technique (convenience sampling) from patients at the Medical Clinic of Gynecology and Perinatology, Gynecology and Obstetrics Hospital, Poznan University of Medical Sciences. The control group was recruited during the checkups in the Outpatient Clinic of the Department of Developmental Gynecology and Sexology, Poznań University of Medical Sciences. The anthropometric and metabolic parameters of the patients were described in [31]. This research was conducted in accordance with the Helsinki Declaration and approved by the Bioethics Committee of the Poznań University of Medical Sciences (approval no. 553/18, addendum no. 161/20). All participating girls and their parents signed informed consent prior to the study. This trial was registered as NCT04738409 at the Clinical Trials Registry (http://www.clinicaltrials.gov (accessed on 4 February 2021).

### 2.2. Medical and Biochemical Evaluation

Hormone levels and other biochemical parameters were measured between 7 and 9 a.m. after overnight fasting, between the 3rd and 5th day of the menstrual cycle (follicular phase). Total testosterone (tT), dehydroepiandrosterone-sulfate (DHEA-S), sex hormone binding globulin (SHBG) were measured by the electrochemiluminescence (ECLIA) immunoassay method (Elecsys; Roche Diagnostics GmbH, Mannheim, Germany) according to the manufacturer’s instructions. The free testosterone (fT) was calculated from tT and SHBG levels using the online fT calculator available on the ISSAM website (www.issam.ch/freetesto.htm (accessed on 17 October 2021), as described previously [32,33,34]. Serum concentrations of androstenedione (A) were measured by using the DRG Androstenedione ELISA kit (enzyme linked immunosorbent assay). The kit uses polyclonal antibodies directed against the antigenic determinants of the analyte being analyzed. The analyses were performed at the Chair and Department of Medical Chemistry and Laboratory Medicine, Poznań University of Medical Sciences.

A pelvic examination with ultrasound and anthropometric assessment (body weight and height) was carried out on all participating girls, supplemented by the determination of the pubertal stage (according to Tanner) and a structured hirsutism assessment using the Ferriman–Gallwey Score.

### 2.3. Evaluation of Macronutrients Intake and PA

The evaluation of eating habits was performed by use of the current quotation method: 3-day food record, described previously [32,35]. The comparison of dietary patterns and selected macronutrients intake (saturated fatty acids (SFA), monounsaturated fatty acids (MUFA), polyunsaturated fatty acids (PUFA), cholesterol, total and plant protein, carbohydrates including fiber) in PCOS patients and healthy controls was presented elsewhere [31].

“Work/school time” and “leisure time” PA were assessed using the Beliefs and Eating Habits Questionnaire (KomPAN), developed and validated by the Commission of Behavioral Determinants of Nutrition from the Polish Academy of Sciences (Warsaw, Poland) (33). According to the KomPAN questionnaire, the PA in each domain was finally defined at one of the three levels: low, moderate and high. For leisure time PA, “low” was described as “sedentary lifestyle, watching TV, reading the press and books, light housework, taking a walk for 1–2 h a week”; “moderate” was described as “walks, cycling, gymnastics, gardening or other light PA performed for 2–3 h a week”; and “high” was described as “cycling, running, working on a plot or garden and other sports activities requiring physical effort, taking up more than 3 h a week”. For work/schooltime PA, “low” means “over 70% of the time in a sitting position”, “moderate” means “approximately 50% of the time in a sitting position and about 50% of the time moving”, and “high” means “about 70% of the time in motion or doing physical work associated with much effort”. The method was fully described in [31,36].

### 2.4. Statistical Analysis

The PQStat v.1.8.2 (PQStat Software Company, Poznań, Poland) software was used for statistical analysis. Descriptive statistics are presented as median and lower and upper quartiles or mean and standard deviation (SD). Normal distribution of data was checked using the Shapiro–Wilk test. For the comparison of two groups, the unpaired *t*-test and Mann–Whitney test were used, depending on data type. When comparing more than two groups, the Kruskal–Wallis test by ranks with Dunn’s post hoc test were used for data that were not normally distributed. Dietary nutrient intake was adjusted for total energy intake (per 100 kcal). The Spearman’s and Pearson’s correlation coefficients were calculated when analyzing correlations between variables. All results were considered as statistically significant when *p* ≤ 0.05.

## 3. Results

**Figure 1:**
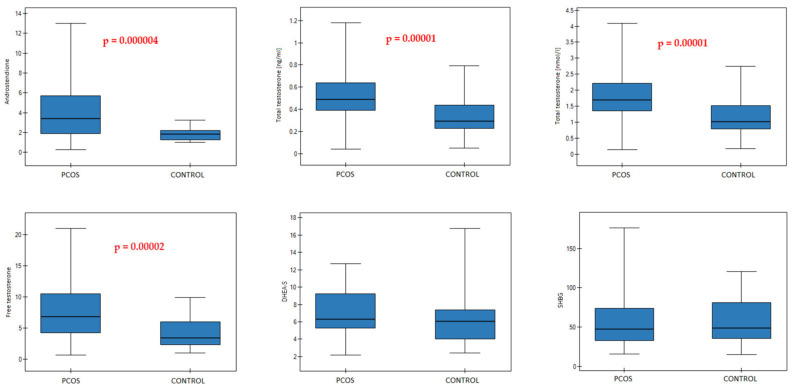
Median (25–75%) values of sex hormones in PCOS and CONTROL groups. *p* values are included when hormone concentrations significantly differed between the two groups.

The anthropometric, physical activity and dietary patient characteristics were described in our previous publication (Tables 1 and 2 in [31]). The PCOS group differed significantly from the control group according to the A (*p* = 0.000004), fT (*p* = 0.00002), and tT serum concentration levels (*p* = 0.00001). There were no significant differences between the groups in serum concentrations of DHEA-S and SGBG (Table 1, Figure 1).

Table 2 presents correlations between hormonal parameters and macronutrients intake adjusted for total energy intake (per 100 kcal). Within the entire study population (both study and control groups), a significant correlation was found between the androstenedione serum concentration and total fat, as well as MUFA intake (*p* = 0.01, *r* = 0.25; *p* = 0.02, *r* = 0.24, respectively). Total protein, plant protein, and fiber intake inversely correlated with androstenedione (*p* = 0.02, *r* = −0.23; *p* = 0.009, *r* = −0.27; *p* = 0.002, *r* = −0.31), tT (*p* = 0.002, *r* = −0.32; *p* = 0.04, *r* = −0.21; *p* = 0.009, *r* = −0.27, respectively). Moreover, plant protein, fiber and carbohydrate intake inversely correlated with fT (*p* = 0.02, *r* = −0.23; *p* = 0.004, −0.29; *p* = 0.04, *r* = −0.21, respectively). Significant correlations could be demonstrated for fT and tT and total fat (*p* = 0.004, *r* = 0.29; *p* = 0.015, *r* = 0.25, respectively), and for fT and MUFA, and PUFA (*p* = 0.02, *r* = 0.24; *p* = 0.05, *r* = 0.21, respectively). No correlation was found between SHGB and DHEA-S, as well as macronutrients intake (Table 2).

“Work/school” PA and “leisure-time” PA inversely correlated with A, total T, and free T. A positive correlation was found between SHBG and PA during leisure time (Table 2).

Table 3 and Table 4 present correlations between hormonal parameters and macronutrients intake adjusted for total energy intake (per 100 kcal), separately for the PCOS group and healthy controls. Surprisingly, in the PCOS group, no correlation was found (Table 3). In contrast, several significant correlations could be observed in healthy controls, for instance, between SHBG and protein, as well as carbohydrates intake (*p* = 0.02, *r* = 0.42; *p* = 0.03, *r* = −0.38, respectively). Furthermore, significant associations could be observed between tT and total protein intake (*p* = 0.007, *r* = −0.45), and fT and total fat, as well as SFA intake (*p* = 0.03, *r* = 0.38; *p* = 0.01, *r* = 0.42, respectively) (Table 4).

Contrary to the associations between macronutrients intake and hormones, numerous correlations between both domains of PA and serum androgens or SHBG could be observed solely in the PCOS group. As shown in Table 3, both PA at work/school and PA during leisure time were negatively correlated with A and free T, and positively correlated with SHBG. No correlations were found between serum androgen levels and PA within the control group (Table 4).

A detailed analysis of all significant correlations observed in the PCOS subgroup as well as in the entire study population was performed in regard to different levels (low, medium, high) and categories (work/school vs. leisure time) of PA. In girls with PCOS, serum concentrations of fT, A and SHBG differed significantly between those reporting “low” versus “high” leisure time PA. As shown in Table 5, PCOS patients with a low level of leisure time activities were characterized by significantly higher mean serum concentrations of A (5.2 vs. 2.01 ng/mL) and free testosterone (8.35 vs. 4.35 ng/mL), and significantly lower SHBG levels (34.83 vs. 67.86 nmol/L).

**Figure 2:**
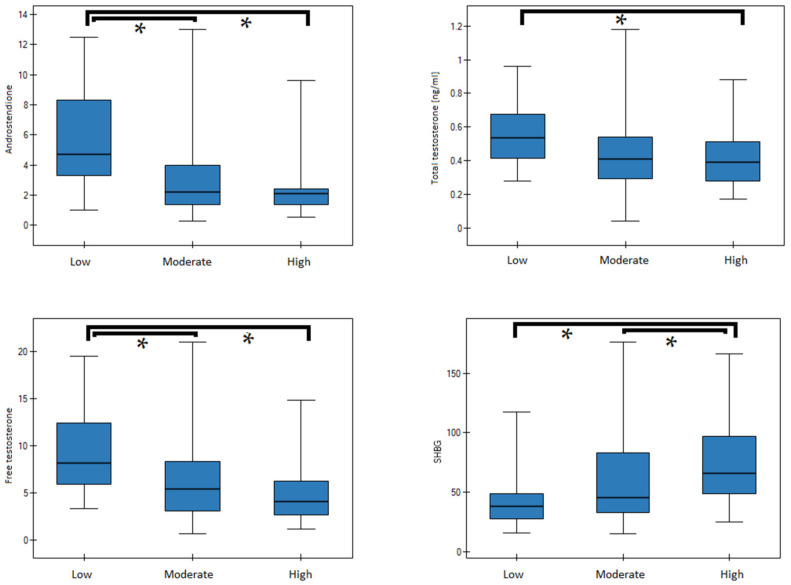
Median (25–75%) of sex hormone concentrations in groups with low, moderate or high PA (leisure). Significant differences are marked by an asterisk (*).

**Figure 3:**
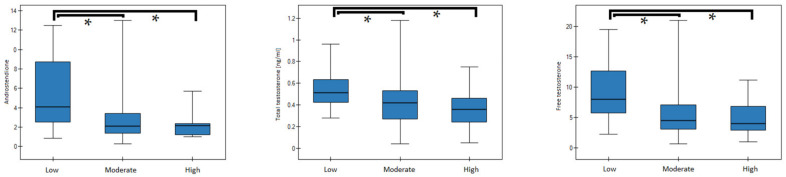
Median (25–75%) of sex hormone concentrations in groups with low, moderate or high PA (work/school). Significant differences are marked by an asterisk (*).

In the entire study cohort (patients and controls), significant differences of fT, A and SHBG concentrations were noticed between subgroups with “low” and “high”, as well as “low” and “moderate” leisure time PA. Additionally, patients reporting “low” versus “high” PA demonstrated significant differences of tT (see Table 6, Figure 2). In regard to “work/school” PA, significant differences in the serum concentrations of A, tT, and fT between girls with low PA and those with moderate or high PA (Table 6, Figure 3).

## 4. Discussion

The role of dietary modifications and PA has been investigated in women with PCOS; however, this has mainly been researched with a focus on weight reduction and improving IR [21,23,24,25,26,39]. It is not yet clear which levels of PA and which concentrations of dietary macronutrients are relevant to androgen or SHBG concentrations. Studies addressing the above questions in adolescent patients are rare and equivocal. Our work provides evidence that there are associations between serum androgen concentrations, macronutrients intake (adjusted for total energy intake per 100 kcal) and PA, but also demonstrates remarkably different patterns of these relationships in girls with and without PCOS. The most striking finding of our study is that macronutrients influenced circulating androgens predominantly in healthy girls. In contrast, the effects of different levels and types of PA were observed almost exclusively in PCOS girls. Finally, we could not observe any association between macronutrients intake, PA and circulating DHEA-S levels.

### 4.1. Relationship between Fat Intake and Androgen Status

We observed several associations between androgen levels (A, tT, fT) and total fat, SFA, PUFA and MUFA intake in healthy girls. This effect also remained statistically significant for the entire cohort but disappeared completely when it was restricted to PCOS patients. For example, fT showed a positive correlation with total fat, MUFA and PUFA in the entire cohort, and—additionally—with SFA and total fat intake in the control group.

The regulatory effects of dietary fats on circulating androgens in women are under-reported. However, an overview of the available studies suggests at least similar pathophysiological mechanisms. Low-fat diets appear to decrease testicular T production and circulating T levels in men, especially those of European ancestry [40]. Wang et al. precisely calculated that a reduction in dietary fat intake (and increase in fiber) resulted in 12% lower circulating androgen levels without changing the clearance [41]. A symmetrical effect was reported by Dorgan et al. [42], who observed an increase in serum and urinary T levels during a high-fat, low-fiber diet in healthy men [42]. According to Mai et al., free fatty acids (FFAs) increase the synthesis of androgen precursors (DHEA and androstenedione) in vivo in men [43]. Furthermore, the same authors performed a randomized controlled cross-over trial providing evidence that FFAs increase adrenal androgen precursors also in healthy young women, independently of a subsequent IR [44]. They suggested that comparable mechanisms might be relevant to the regulation of androgen synthesis in men as well as to the development of hyperandrogenemia in women with PCOS [44].

This hypothesis is consistent with observations in women that a high-fat diet rich in SFA increases IR and metabolic disorders amongst patients suffering from overweight and PCOS [45,46], but contrasting to, e.g., reports of a decrease in T levels after a high-fat, Western meal compared to a low-fat, high-fiber meal, and increased glucose and insulin levels after a low-fat, high-fiber meal in women with PCOS [47]. However, we interpret these one-dimensional observations with caution. Lowering circulating androgens is not an isolated therapeutical goal in PCOS, and postprandial fluctuations of isolated hormones are not a sufficient explanation for the impact of diet on the development and course of a multifaceted endocrinopathy such as PCOS.

Although we did not observe any significant associations between serum androgens and fat consumption in PCOS girls in the present study, our previous data (obtained in the same study population) identified the high fat consumption, including SFA, MUFA and PUFA, as a strong predictor of PCOS, increasing the chance to be diagnosed with PCOS up to threefold [31]. These observations are not contradictory, given the complex and still not fully elucidated etiology of PCOS. In our opinion, the role of dietary fats on the hormonal and metabolic profile in PCOS should be analyzed separately for SFA and unsaturated fatty acids (MUFA and PUFA), as well as in regard to n-3 PUFA and n-6 PUFA. Unfortunately, the relationship between MUFA and PUFA intake and androgen (especially T) concentrations in patients with PCOS has been inconsistently reported [26,48]. This ambivalence is reflected in the most recent analysis (as of 2021) including 10 randomized controlled trials with 610 patients, which showed that the supplementation of n-3 PUFAs in PCOS women can significantly improve, among others, the levels of C-reactive protein (CRP), tT, luteinizing hormone and SHBG, but did not affect the concentrations of DHEAS, free androgen index or follicle-stimulating hormone [49]. Furthermore, antithetical effects of MUFA and PUFA on T levels was described by Barrea et al., with an inverse correlation between MUFA intake and T levels, and a strong positive correlation between dietary PUFAs and T [50].

Previously, we reported a significantly higher PUFA and MUFA intake in PCOS patients as compared to healthy controls [31]. Notwithstanding this, our present evaluation failed to demonstrate any correlation of PUFA or MUFA intake with the levels of circulating androgens, an effect which was mostly apparent in the entire (mixed) cohort and persisted (limited to tT) in healthy controls. Our actual findings are in accordance with the results of a small intervention study of Kasim-Karakas et al. [51], showing that in women with PCOS a partial replacement of dietary fats with PUFAs for the period of three months did not affect neither tT, nor DHEA-S nor SHBG levels. Nonetheless, a PUFA-rich diet led to unfavorable metabolic effects as evidenced by elevated fasting glucose and worsened area under the curve for glucose during oral glucose tolerance test [46]. Lastly, a meta-analysis assessing the effect of n-3 PUFAs on adult females with PCOS provided—after an evaluation of eight clinical trials with a total of 298 participants—evidence that n-3 PUFA supplementation does not seem to affect the androgenic profile of adult females with PCOS, although some studies reported marginal reductions in tT or DHEAS levels [52]. In the group of healthy controls—in contrast to PCOS patients—the intake of total fat and SFA was positively correlated with fT levels. As expected, the associations of PUFA, MUFA and total fat consumption with hormonal parameters persisted in the entire (mixed) study population. These observations are of importance and require further investigation. We hypothesize that the disparities in the results of studies focusing on dietary fats can partially result from not being able to distinguish between the independent effects of n-3 PUFA and n-6 PUFA, their relative proportions in the diet, modulating action of other macronutrients or role of co-existing hormonal or metabolic disturbances (e.g., IR).

### 4.2. Relationship between Protein, Carbohydrates and Fiber Intake and Androgene Status

In healthy girls, we observed significant, adverse, and strong associations of tT with the consumption of total protein, but not with plant protein intake. Total protein was, in turn, positively correlated with SHGB levels. Interestingly, a weak, adverse association of carbohydrate consumption with SHBG levels was found.

This finding is interesting from a pathophysiological and therapeutical point of view. In patients with PCOS, a modest reduction in dietary carbohydrates has been supposed to lower both circulating testosterone and insulin [53]. Of note, excess or the dysregulation of both hormones are of key importance in PCOS. Similarly, Douglas et al. [54] demonstrated that eucaloric low-carbohydrate diet reduced the fasting and postchallenge insulin concentrations among women with PCOS [54]. A small pilot study of Mavropoulos et al. [55] reported significant reductions in fT, LH/FSH ratio, and fasting insulin in women with obesity and PCOS applying a low-carbohydrate, ketogenic diet. Interventions helping to overcome the IR in PCOS patients, are essential also for normalization of hyperandrogenemia [56], since insulin resistance, inducing compensatory hyperinsulinemia, induces several pathways finally leading to a subsequent excess of circulating androgens [57]. Mehrabani et al. observed a beneficial effect on testosterone and insulin levels, homeostatic model assessment for insulin resistance (HOMA), and high-sensitivity C- reactive protein concentrations after 12 weeks of a high-protein, low-glycemic load diet provided to overweight and obese women with PCOS [58].

Studies focusing on the relationship between dietary fiber intake and androgen levels in women are inconsistent. In the report of Cutler et al. [59], fiber intake was inversely correlated with IR, fasting insulin, glucose tolerance, T, and DHEA-S in middle-aged women with PCOS. Contrary to these findings, our study could not identify any specific association between fiber intake and androgen concentration in women with PCOS, but otherwise showed adverse correlation between fiber intake and tT, fT, and A in entire cohort. We consider both findings to be relevant, especially from the pathophysiological point of view. We therefore postulate a more differentiated approach to this topic in future research, e.g., by evaluating the effects of fiber intake on androgen levels in relation to selected plant products (cereals, vegetables and fruits, nuts, legumes, etc.) and to varying proportions of soluble and insoluble forms in those products. Indeed, both types of fiber differ according to their metabolic effects, eventually also to their impact on circulating androgens. We also hypothesize that the impact of macronutrients such as fiber or protein could be modulated by the glycemic index (GI) of particular products. This hypothesis is supported by our earlier observation that PCOS patients preferably consumed high and medium glycemic index (GI) foods [31]. Moreover, we demonstrated that the consumption of products with medium GI increased the likelihood to be diagnosed with PCOS by threefold [31]. Unlike products with low GI, products with high and medium GI usually contain only few fiber and complex carbohydrates [60], resulting in their different effects on glucose and insulin levels and eventually on androgen levels [53,57].

In healthy girls, we demonstrated a positive correlation of total protein intake with SGBG concentrations, but not with the consumption of plant protein. This observation partially contrasts with the study of Moran et al. [61], who report an increase in the free androgen index (calculated as tT-to-SHBG ratio) by 44% in response to low-protein diet in PCOS women.

Similar to our conclusions regarding fat intake, the lack of association between (total or plant-derived) protein intake and androgen levels in adolescents with PCOS does not automatically imply a lack of importance of protein, carbohydrate or fiber intake in the pathophysiology of PCOS. We assume that in a pathological condition such as PCOS, numerous hyperandrogenemia-inducing mechanisms (e.g., IR, hypo-SHBG-emia, under-reported roles of systemic and ovarian renin-aldosterone-renin system) simply “overtrump” the impact of diet-related factors. In this context, we refer to our earlier observations indicating an inverse correlation between plant protein intake and fasting glucose, fasting insulin and the HOMA-IR index [31]. Furthermore, the content of dietary fiber and protein intake, mainly of plant origin, correlated negatively with markers of inflammation and oxidative stress, especially in overweight and obese girls with PCOS [35]. Finally, an increase of 10 g in plant protein intake decreased the probability of being overweight and of obesity in adolescent diagnosed with PCOS [32].

### 4.3. Relationship between PA and Androgen Status

In our present work, we demonstrated that in adolescents with PCOS the “work/school” PA and “leisure” PA were both negatively correlated with serum androgen (A and fT) concentrations and positively associated with SHBG levels. This observation is potentially of paramount therapeutical importance for adolescent patients. Nevertheless, confirmation from long-term and randomized studies is urgently warranted.

A further stratification of PA levels (low-medium-high) revealed lower A and fT levels in patients reporting high or moderate PA, and—on the contrary—higher androgene concentrations in girls with low PA. The opposite association occurred between PA and SHBG. These results are in accordance with our previous findings, identifying moderate or high PA levels (both “work/school” PA and “leisure-time” PA) as negative predictors for PCOS, independently of age and BMI [31].

Nevertheless, several studies questioned causal relationships of energy intake, nutrient composition, diet quality and PA with PCOS [62,63,64], concluding that recommendations regarding diet and PA for PCOS patients should not differ from those for the general population [62]. However, the need for longitudinal studies to assess the influence of diet and PA on the development and/or the progression of PCOS was emphasized in order to establish a causal relationship [62].

Eventually, the dependence of T levels from intensity of physical exercise has been more frequently studied in men than in women [65,66,67]. A meta-analysis of randomized and controlled trials, uncontrolled trials and control trials confirmed the exercise-dependent, albeit slight, increase in salivary T and cortisol levels in males. It is noteworthy that the effect sizes were influenced by the type of training (aerobic, resistance and power exercise) and the study design [66,67].

## 5. Strengths and Limitations

The major strength of our study is, that—to the best of our knowledge—we are the first to report on the associations between circulating androgens, dietary macronutrients and PA in adolescent girls. Nevertheless, our study has some limitations. Firstly, we were not able to obtain data concerning specific sources of dietary fiber and PUFA, which would be necessary for more far-reaching conclusions concerning the optimal diet of girls with PCOS. Secondly, our results do not allow us to draw conclusions regarding the therapeutic relevance, because of their observational, and not interventional, nature. A further shortcoming of our study is the lack of data concerning insulin concentrations and other parameters of glucose metabolism, since insulin is involved in androgen regulation and IR remains one of the hallmarks of PCOS. As a final caveat we consider the size of the control group and the fact that only convenience sampling was used.

## 6. Conclusions

We observed several associations between dietary components (total fat, SFA, total protein) and concentrations of circulating androgens in healthy women but not participants affected by PCOS. We did not observe any significant correlations between DHEA-S concentrations and dietary macronutrients intake.

Inverse correlations between PA and androgen concentrations and positive correlations with SHBG were observed only in PCOS patients. Moreover, the concentration of androgens in these women varied with different levels of PA. We hypothesize that the latter observations could have therapeutic implications. Further research should focus on identifying optimal PA levels and nutrition models in randomized trials, that could be recommended by family doctors, pediatricians, and gynecologists in the non-pharmacological treatment of young patients with PCOS.

## Figures and Tables

**Table 1:** Hormonal characteristics of the PCOS and CONTROL groups.

	PCOS *n* = 61	CONTROL *n* = 35	*p*-Value
Androstenedione (ng/mL)			*0.000004*
	3.4 (1.9–5.7)	1.85 (1.26–2.21)	
Total T (ng/mL)			*0.00001*
	0.49 (0.39–0.64)	0.29 (0.23–0.44)	
Total T (nmol/L)			*0.00001*
	1.7 (1.35–2.22)	1.01 (0.78–1.51)	
Free T (ng/L)			*0.00002*
	6.8 (4.2–10.5)	3.38 (2.34–6)	
DHEA-S (μmol/L)			0.052
	6.32 (5.31–9.25)	6.05 (4.05–7.39)	
SHBG (nmol/L)			0.648
	47.55 (32.96–73.8)	48.96 (35.29–81.38)	

Data presented as median (25–75%). Abbreviations: Total T—total testosterone; Free T—free testosterone; DHEA-S—dehydroepiandrosterone; SHBG—sex hormone-binding globulin; all the *p*-values marked in italics are statistically significant. Serum reference ranges in adolescent girls are: for Androstenedione: 3.1–10.9 nmol/L (0.97–3.43 ng/mL) [37], Total T: <51 ng/dL (<0.51 ng/mL) [38] and for DHEA-S: 1.77–9.99 μmol/L, and SHBG: 26.1–110 nmol/L—according to the hospital age- and sex-specific laboratory reference ranges.

**Table 2:** Correlations among serum androgen level, and macronutrient intake adjusted for total energy intake (per 100 kcal) and PA in the whole study population (PCOS + CONTROL group) ^T^.

Variables	Total Protein [g/100 kcal]	Total Fat [g/100 kcal]	Carbohydrates [g/100 kcal]	Fiber [g/100 kcal]	Plant Protein [g/100 kcal]	SFA [g/100 kcal]	MUFA [g/100 kcal]	PUFA [g/100 kcal]	Cholesterol [mg/100 kcal]	PA (Work/School)	PA (Leisure Time)
A *p*-value	*0.024*	*0.013*	0.118	*0.002*	*0.009*	0.324	*0.017*	0.051	0.403	*0.0004*	*0.0003*
*r*	−0.231	0.253	−0.160	−0.313	−0.266	0.102	0.244	0.200	−0.086	−0.359	−0.365
Total T *p*-value	*0.002*	*0.015*	0.194	*0.009*	*0.042*	0.233	0.138	0.128	0.263	*0.001*	*0.015*
*r*	−0.319	0.248	−0.134	−0.266	−0.208	0.123	0.152	0.156	−0.115	−0.333	−0.248
Free T											
*p*-value	0.111	*0.004*	*0.039*	*0.004*	*0.024*	0.143	*0.020*	*0.045*	0.925	*0.001*	*0.0002*
*r*	−0.164	0.293	−0.211	−0.288	−0.230	0.150	0.238	0.205	0.010	−0.344	−0.375
DHEA-S *p*-value	0.126	0.569	0.974	0.868	0.505	0.820	0852	0.382	0.745	0.173	0.381
*r*	−0.157	0.059	−0.003	−0.017	−0.069	−0.024	0.019	0.090	−0.034	−0.143	−0.090
SHBG *p*-value	0.100	0.680	0.830	0.245	0.539	0.558	0.243	0.129	0.403	0.094	*0.0005*
*r*	0.169	−0.043	−0.022	0.120	0.063	0.060	−0.120	−0.156	0.086	0.175	0.351

Abbreviations: A—androstenedione; Total T—total testosterone; Free T—free testosterone; DHEA-S—dehydroepiandrosterone; SHBG—sex hormone-binding globulin; SFA—saturated fatty acid; MUFA—monounsaturated fatty acids; PUFA—polyunsaturated fatty acids; PA—physical activity; the *r* coefficients are Spearman’s correlation coefficients. All the *p*-values marked in italics are statistically significant. ^T^ Data in this table including the same participants in this research, were previously partly presented at the 1st International Electronic Conference on Clinical Medicine.

**Table 3:** Spearman’s correlation coefficients among serum androgen levels and macronutrient intake adjusted for total energy intake (per 100 kcal) and PA (PCOS group).

Variables	Total Protein [g/100 kcal]	Total Fat [g/100 kcal]	Carbohydrates [g/100 kcal]	Fiber [g/100 kcal]	Plant Protein [g/100 kcal]	SFA [g/100 kcal]	MUFA [g/100 kcal]	PUFA [g/100 kcal]	Cholesterol [mg/100 kcal]	PA (Work/School)	PA (Leisure Time)
A *p*-value	0.425	0.359	0.547	0.069	0.141	0.874	0.164	0.605	0.643	*0.029*	*0.001*
*r*	−0.104	0.120	−0.079	−0.235	−0.191	−0.021	0.180	0.068	−0.062	−0.282	−0.404
Total T *p*-value	0.415	0.497	0.599	0.064	0.079	0.700	0.703	0.404	0.515	0.183	0.132
*r*	−0.106	0.089	−0.069	−0.239	−0.227	0.050	0.050	−0.109	−0.085	−0.174	−0.195
Free T *p*-value	0.436	0.387	0.556	0.059	0.100	0.943	0.257	0.862	0.660	*0.045*	*0.003*
*r*	−0.102	0.113	−0.077	−0.243	−0.213	−0.009	0.147	0.023	−0.057	−0.260	−0.374
DHEA-S *p*-value	0.660	0.932	0.871	0.938	0.225	0.782	0.797	0.615	0.638	0.899	0.899
*r*	−0.058	0.011	0.021	−0.010	−0.148	−0.036	−0.034	−0.066	0.061	0.017	−0.017
SHBG *p*-value	0.734	0.229	0.372	0.132	0.264	0.880	0.062	0.196	0.892	*0.018*	*0.0002*
*r*	0.044	−0.156	0.116	0.195	0.145	0.020	−0.240	−0.168	0.018	0.304	0.457

Abbreviations: A—androstenedione; Total T—total testosterone; Free T—free testosterone; DHEA-S—dehydroepiandrosterone; SHBG—sex hormone-binding globulin; SFA, saturated fatty acid; MUFA, monounsaturated fatty acids; PUFA, polyunsaturated fatty acids; PA—physical activity; the *r* coefficients are Spearman’s correlation coefficients. All the *p*-value marked in italics were statistically significant.

**Table 4:** Spearman and Pearson’s correlation coefficients among serum androgen level, and macronutrient intake and PA. (CONTROL group).

Variables	Total Protein [g/100 kcal]	Total Fat [g/100 kcal]	Total Carbohydrates [g/100 kcal]	Fiber [g/100 kcal]	Plant Protein [g/100 kcal]	SFA [g/100 kcal]	MUFA [g/100 kcal]	PUFA [g/100 kcal]	Cholesterol [mg/100 kcal]	PA (Work/School)	PA (Leisure Time)
A *p*-value	0.123	0.074	0.458	0.546	0.791	0.708	0.534	0.404	0.676	0.895	0.193
*r*	−0.265	0.305	−0.130	−0.106	−0.047	0.066 *	0.109	0.146	0.073	0.024	0.225
Total T *p*-value	*0.007*	0.467	0.612	0.835	0.527	0.708	0.748	0.223	0.896	0.319	0.997
*r*	−0.448 *	0.127 *	0.089 *	−0.037	0.111 *	0.066 *	0.056	0.211	−0.023	−0.179	0.001
Free T *p*-value	0.544	*0.026*	0.135	0.491	0.794	*0.013*	0.452	0.403	0.116	0.665	0.651
*r*	−0.106	0.375	−0.258	−0.120	0.046	0.417	0.131	0.146	0.270	−0.078	−0.079
DHEA-S *p*-value	0.284	0.607	0.322	0.235	0.173	0.707	0.117	0.331	0.512	0.190	0.686
*r*	−0.186	−0.090	0.173	0.206	0.236	−0.066	−0.270	0.169	−0.115	−0.234	−0.071
SHBG *p*-value	*0.022*	0.196	*0.025*	0.607	0.244	0.294	0.152	0.844	0.240	0.579	0.394
*r*	0.387	0.224	−0.379	−0.090	−0.202	0.182	0.247	−0.034	0.204	−0.100	0.149

Abbreviations: A—androstenedione; Total T—total testosterone; Free T—free testosterone; DHEA-S—dehydroepiandrosterone; SHBG—sex hormone-binding globulin; SFA, saturated fatty acid; MUFA, monounsaturated fatty acids; PUFA, polyunsaturated fatty acids; the *r* coefficients are Spearman correlation coefficients (not marked) or Pearson’s correlation coefficients (marked by an asterisk). All the *p*-value marked in italics were statistically significant.

**Table 5:** Comparison of the serum concentrations of A, fT and SHBG depending on the level of PA (PCOS group).

PA (Leisure)	A (ng/mL)	*p*-Value	Dunn–Bonferroni	Low	Moderate	High
low	5.20 (3.40–8.65)	*0.007*	low		0.221	*0.005*
moderate	3.60 (1.85–5.65)		moderate	0.221		0.269
high	2.01 (1.66–3.07)		high	*0.005*	0.269	
**PA (leisure)**	Free T (ng/mL)	*p*-value	Dunn–Bonferroni	Low	moderate	high
low	8.35 (7.05–13.03)	*0.015*	low		0.298	*0.011*
moderate	6.60 (4.15–9.45)		moderate	0.298		0.339
high	4.35 (3.93–6.45)		high	*0.011*	0.339	
**PA (leisure)**	SHBG (nmol/L)	*p*-value	Dunn–Bonferroni	Low	moderate	high
low	34.83 (27.01–45.95)	*0.002*	low		0.137	*0.001*
moderate	47.55 (35.32–85.92)		moderate	0.137		0.154
high	67.86 (57.15–107.15)		high	*0.001*	0.154	

Abbreviations: A—androstenedione; Free T—free testosterone; Total T—total testosterone; SHBG—sex hormone-binding globulin; PA—physical activity; all the *p*-value marked in italics were statistically significant.

**Table 6:** Comparison of the concentration of sex hormones depending on the level of PA in the whole study population (PCOS + CONTROL group).

PA (Work/School)	A	*p*-Value	Dunn–Bonferroni	Low	Moderate	High
low	4.10 (2.51–8.70)	*0.001*	low		*0.004*	*0.006*
moderate	2.10 (1.39–3.40)		moderate	*0.004*		1.000
high	2.14 (1.22–2.34)		high	*0.006*	1.000	
**PA (work/school)**	Total T	*p*-value	Dunn–Bonferroni	Low	moderate	high
low	0.51 (0.43–0.64)	*0.005*	low		*0.036*	*0.008*
moderate	0.42 (0.27–0.53)		moderate	*0.036*		0.729
high	0.36 (0.24–0.46)		high	*0.008*	0.729	
**PA (work/school)**	Free T	*p*-value	Dunn–Bonferroni	Low	moderate	high
low	8.00 (5.70–12.70)	*0.002*	low		*0.006*	*0.009*
moderate	4.50 (3.09–7.08)		moderate	*0.006*		1
high	4.00 (2.93–6.80)		high	*0.009*	1	
**PA (leisure)**	A	*p*-value	Dunn–Bonferroni	Low	moderate	high
low	4.70 (3.33–8.30)	*0.0009*	low		*0.014*	*0.001*
moderate	2.19 (1.36–4.00)		moderate	*0.014*		0.610
high	2.09 (1.39–2.44)		high	*0.001*	0.610	
**PA (leisure)**	Total T	*p*-value	Dunn–Bonferroni	low	moderate	high
low	0.54 (0.41–0.68)	*0.03*	low		0.087	*0.033*
moderate	0.41 (0.29–0.54)		moderate	0.087		1
high	0.39 (0.28–0.51)		high	*0.033*	1	
**PA (leisure)**	Free T	*p*-value	Dunn–Bonferroni	Low	moderate	high
low	8.15 (5.93–12.38)	*0.0009*	low		*0.027*	*0.001*
moderate	5.40 (3.10–8.30)		moderate	*0.027*		0.339
high	4.10 (2.68–6.25)		high	*0.001*	0.339	
**PA (leisure)**	SHBG	*p*-value	Dunn–Bonferroni	Low	moderate	high
low	38.07 (27.52–48.39)	*0.003*	low		0.448	*0.002*
moderate	45.28 (33.13–83.17)		moderate	0.448		*0.049*
high	66.11 (48.96–96.68)		high	*0.002*	*0.049*	

Abbreviations: A—androstenedione; Free T—free testosterone; SHBG—sex hormone-binding globulin; PA—physical activity; all the *p*-value marked in italics were statistically significant.

## Data Availability

The datasets may be available from the corresponding author on reasonable request.
